# Influence of life intervention on anxiety, depression, and quality of life of COVID-19 patients: A protocol for systematic review and meta-analysis

**DOI:** 10.1097/MD.0000000000025391

**Published:** 2021-05-07

**Authors:** Yuli Qian, Huan Xu, Jing Diao, Qiaozhen Li, Qian Zhan, Yujiao Fang

**Affiliations:** a Department of Neurology, The Third People's Hospital of Hubei Province Affiliated to Jianghan University, Wuhan 430000, Hubei Province; b Department of Geriatrics, West China Hospital, Sichuan University, Chengdu 610041, Sichuan Province, China.

**Keywords:** anxiety, coronavirus disease 2019, depression, life intervention, systematic review

## Abstract

**Background:**

Coronavirus disease 2019 (COVID-19) patients suffer from anxiety, depression, and sleep disorder due to isolation treatment and other reasons. Whether life interventions can be an alternative therapy for COVID-19 patients, accompanied with anxiety, depression, and sleep disorder, is controversial. Therefore, we conducted a meta-analysis and systematic review to evaluate the effects of life interventions on anxiety, depression, and sleep disorder in COVID-19 patients to provide some guidance for clinical application.

**Methods:**

The randomized controlled trials related to the life intervention and COVID-19 from inception to February 2021 will be searched. The following databases are our focused areas: the Cochrane Central Register of Controlled Trials, PubMed, MEDLINE, EMBASE, Web of Science, China National Knowledge Infrastructure, Chinese Biomedical Literature Database, and Wan Fang Database. Two investigators would independently screen the literature according to the inclusion and exclusion criteria, extract data, and evaluate the risk of bias in the included studies. Meta-analysis was performed with RevMan 5.3 software.

**Results:**

The results will provide a high-quality synthesis of current evidence for researchers in this subject area.

**Conclusion:**

The conclusion of our study will provide evidence for the judgment of whether life intervention is an effective intervention on COVID-19 patients.

**PROSPERO registration number:**

CRD42020199802.

## 1 Introduction

Coronavirus disease 2019 (COVID-19) is a new coronavirus that has never been discovered before,^[1–3]^ and it can cause rapid SARS and spread worldwide.^[4,5]^ It is manifested with fever or chills, cough, shortness of breath, fatigue, muscle or body soreness, sore throat, congestion or runny nose, and difficulty breathing.^[6,7]^ Poor treatment may further give rise to pneumonia, severe acute respiratory syndrome, kidney failure, and even death.^[8,9]^

At present, the main source of infection is COVID-19 infection, dominantly through respiratory droplets and close contact.^[10–12]^ Patients diagnosed with the disease must be treated in isolation. COVID-19 and the closed environment of isolation wards will adversely affect patients^[13]^ psychologically. After hospitalization, patients left the familiar working and living environment, and was isolated and closed, so the original way of life was completely disrupted. In addition, the monotonous life in the isolation ward and the lack of recreational activities caused the psychological disorder of patients.^[14]^ Psychological disorder can lead to low immunity and reduce enthusiasm for treatment, which posed negative impacts on the rehabilitation of the disease.^[15]^ A retrospective study revealed that isolation treatment may cause unexpected mental trauma to patients, and may even result in self-harm behaviors such as suicide, and these effects can still exist 3 years after the release of quarantine.^[16]^ Through clinical observation, many patients developed anxiety, depression, and sleep disorder after isolation treatment. As psychological stresses, anxiety, and depression will cause a series of physiological events and the decline of immunity.^[17,18]^ Treating and nursing patients in isolation ward are mainly aimed at the disease itself, and do not pay enough attention to the physical and mental impacts caused by isolation.^[19,20]^ Improving treatment environment in the isolation area and simulating the normal work and rest rules as far as possible are of great significance in relieving emotional tension, dredging psychological disorder, and promoting disease rehabilitation.^[21]^ With the improvement of COVID-19 condition and the experience of discharged patients, timely life intervention can improve the prognosis, maximize the preservation of function, and improve the life quality and psychological function.^[22]^

At present, there is a lack of evidence-based medicine data on the effects of life intervention on anxiety, depression, and life quality in COVID-19 patients. Therefore, in this study, we aimed to systematically review the effects of life intervention on anxiety, depression, and life quality in COVID-19 patients.

## 2 Methods

### 2.1 Study registration

The systematic review protocol has been registered in PROSPERO, and the registration number is CRD42020199802. The consent of this protocol report is based on the Preferred Reporting Items for Systematic Reviews and Meta-Analyses Protocols Statement Guidelines.^[23]^

### 2.2 Inclusion criteria for study selection

Articles related to the influence of life intervention on COVID-19 patients will be included. Due to language restrictions, we would search for articles in English and Chinese, so as to get a more objective and true evaluation. All articles included are randomized controlled trial (RCT) type articles.

#### 2.2.1 Types of participants

Inclusion criteria:

(1) Patients with confirmed diagnosis of COVID-19;
(2) Patients >65 years old;
(3) Patients >6 months after the onset of other acute diseases;
(4) Patients with a mini-mental state examination score >21;
(5) Patient without COPD or any other respiratory diseases;
(6) Patients with a forced expiratory volume in 1 second >70%.

Exclusion criteria:

(1) Patients with moderate or severe heart disease (Grade III or IV, New York Heart Association);
(2) Patients with severe ischemic or hemorrhagic stroke or neurodegenerative diseases.

#### 2.2.2 Types of interventions

The interventions will be life interventions either alone or combined with other therapies. The categories of intervention are the modification of patients and caregiver behaviors and the home-based environment, and hospital-based environmental control measures. The control group included patients who did not receive the above-mentioned life interventions. If possible, we will assess whether the intervention group and the control group follow the same protocol in other aspects of patients’ care. We will describe the interventions adopted by each institute and included in the study.

#### 2.2.3 Types of outcome measures

Anxiety, depression, life quality, and sleep quality scale.

### 2.3 Data sources

The following electronic databases will be searched from inception to February 2021: the Cochrane Central Register of Controlled Trials, PubMed, MEDLINE, EMBASE, Web of Science, China National Knowledge Infrastructure, Chinese Biomedical Literature Database, and Wan Fang Database. About other sources, we also plan to manually search for the unpublished conference articles and the bibliography of established publications.

### 2.4 Search strategy

The search terms on PubMed are as follows: “lifestyle intervention” or “life intervention” and “lifestyle modification” or “life modification”; COVID-19 (e.g., “Corona Virus Disease 2019” or “Corona Virus”); RCT (“random∗” or “clinical trial”). The combination of Medical Subject Headings (MeSH) and text words will be used. These search terms are summarized in Table 1.

**Table 1:** Search strategy for PubMed.

Number	Search terms
#1	Lifestyle intervention[Title/Abstract]
#2	Life intervention[Title/Abstract]
#3	Lifestyle modification[Title/Abstract]
#4	Life modification[Title/Abstract]
#5	#1 OR #2 OR #3 OR #4
#6	Corona Virus [Title/Abstract]
#7	Corona Virus Disease 2019 [Title/Abstract]
#8	COVID-19 [Title/Abstract]
#9	Novel coronavirus[Title/Abstract]
#10	Novel coronavirus pneumonia[Title/Abstract]
#11	#6 OR #7 OR #8 OR #9 OR #10
#12	Random∗[Title/Abstract]
#13	Clinical trial[Title/Abstract]
#14	#12 OR #13
#15	#5 AND #11 AND #14

### 2.5 Data collection and analysis

#### 2.5.1 Selection of studies

**Figure 1:**
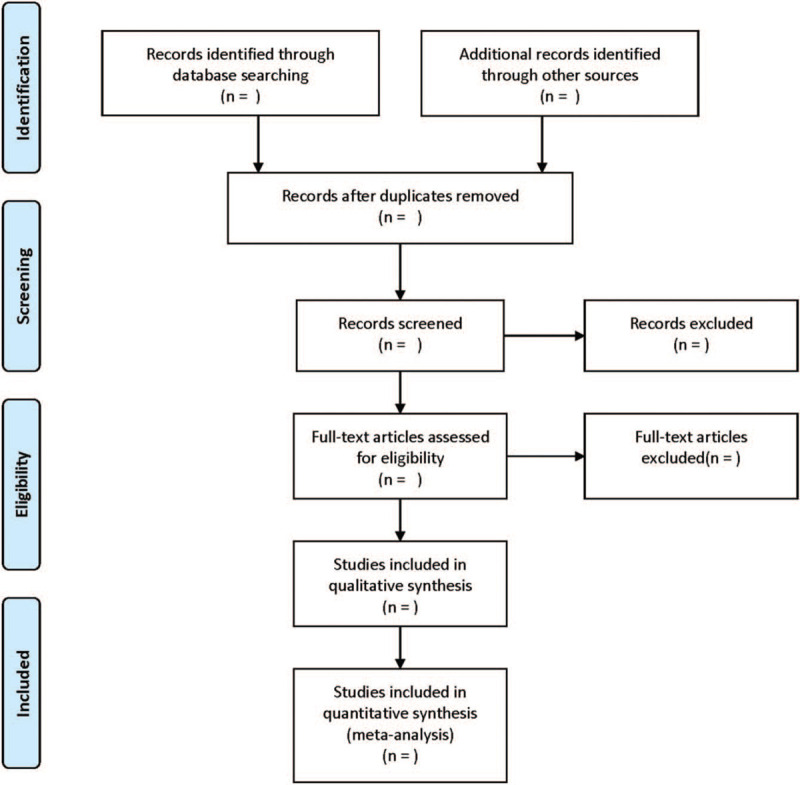
Flow diagram of study selection process.

All reviewers received evidence-based training and adhered to the process summarized. The 2 reviewers independently screened the literature based on the title, abstract, and keywords of literatures, and excluded the irrelevant literatures. The rest of literatures were further confirmed by 2 researchers after reading the full text. The excluded research and the reasons for the exclusion were recorded. The differences between the 2 reviewers were resolved through consensus or by a third independent reviewer. The process of the selection is displayed in Figure 1.

#### 2.5.2 Data extraction and management

Two researchers independently screened the literature and extracted data. If there were differences, a third researcher was asked to judge. Extracted contents include author, publication date, country, sample size, age, type, content and duration of life intervention, type of anxiety, depression, life quality and sleep quality scale, result measurement data, intervention time and details, etc.

#### 2.5.3 Assessment of risk of bias in included studies

The risk of bias assessment method set out in the Cochrane Handbook for Systematic Reviews of Interventions 5.1.0 will be adopted on the included RCTs. The main items to be considered are as follows:

1) randomization plan;
2) group concealment;
3) blinding method;
4) incomplete data reporting;
5) selective outcome report; and
6) other sources of bias.

Each item will be evaluated as “high,” “low,” or “unclear.”

#### 2.5.4 Measures of treatment effects

Standardized mean difference is applied to measure the efficacy of 95% confidence interval.

#### 2.5.5 Unit of analysis issues

We will include data from parallel group design studies for meta-analysis. In these trials, we will collect and analyze individual measurements of each outcome for each participant.

#### 2.5.6 Management of missing data

We will try our best to ensure the integrity of the data. If the included RCT data are not complete, we will try every means to contact the corresponding author of the article by sending emails or making a phone call. If the corresponding author cannot be contacted, we will remove the experiment with incomplete data. After data integrity is assured, intention analysis therapy and sensitivity analysis will be performed.

#### 2.5.7 Assessment of heterogeneity

The heterogeneity included in the results of the study was analyzed by carrying out the *χ*^2^ test (the test level was α = 0.1) and combining with *I*^2^ to quantitatively determine the size of the heterogeneity. When *P* < .1 and/or *I*^2^ > 50%, the random effect model is used for the combined analysis. Otherwise, the fixed-effect model is applied for the combined analysis.

#### 2.5.8 Assessment of reporting biases

Publication bias was evaluated by conducting the funnel plot asymmetry test.

#### 2.5.9 Data synthesis

We will use Review Manager Software (RevMan) V.5.3 (Copenhagen, Denmark) and Stata 14.0 software (Stata Corporation, College Station, TX) for data analysis and quantitative data synthesis. If there are no findings of statistical heterogeneity, the fixed-effect model will be adopted for data synthesis. If there is significant statistical heterogeneity, we will use the random effect model, and all participants will explore the possible causes from a clinical and methodological perspective and provide a descriptive or subgroup analysis.

#### 2.5.10 Subgroup analysis

A subgroup analysis will be carried out on the basis of intervention time (<6 weeks or ≥6 weeks).

#### 2.5.11 Sensitivity analysis

To determine the stability of the outcome measures, each outcome measure was analyzed using sensitivity analysis.

#### 2.5.12 Grading the quality of evidence

According to the Grading of Recommendations Assessment, Development, and Evaluation system, the quality of evidence and the level of recommendation are evaluated.^[24]^ The results of the meta-analysis in RevMan software were imported into Grading of Recommendations Assessment, Development, and Evaluation pro software. The quality of evidence will be divided into high quality, medium quality, low quality, and very low quality.

#### 2.5.13 Ethical review and informed consent of patients

The content of this article does not involve moral approval or ethical review and will be presented in print or at relevant conferences.

## 3 Discussion

COVID-19 is mainly transmitted through the respiratory tract, with the characteristics of strong infectivity and great harmfulness.^[25–27]^ It is a great disaster to individuals, families, and society as well. In order to cut off the route of transmission, it is required to isolate and treat suspected and confirmed cases.^[28]^ In the process of isolation, fear and inferiority caused by infection, worry about the safety of family members, boredom caused by a long isolation period, economic losses caused by the epidemic, closed treatment environment, and so on will have serious impacts on patients psychologically.^[29,30]^ Therefore, there are negative emotions such as anxiety, depression, fear, and so on. Negative psychology seriously affects the recovery of the disease. A good life style and diversified hospital activities are conductive to patients to adapt to the hospital environment, and are of great significance to improve their life quality and promote physical and mental rehabilitation. At present, there is no meta-analysis to the effects of life intervention on anxiety, depression, and life quality in COVID-19 patients. It is hoped that this meta-analysis can provide a convincing scientific basis and guide clinical practices.

This study has some limitations. This study only searches Chinese and English databases, which may be incomplete. In this meta-analysis, there are few studies that can carry out meta-analysis, and the information provided by the literature is also limited. We are unable to analyze the indicators we are concerned about through subgroup analysis. Different life intervention methods, differences in the intervention process, and defects in the experimental design may lead to certain heterogeneity of the results.

To sum up, life intervention for COVID-19 patients may help patients adjust their mentality, reduce their anxiety and depression, and improve their life quality.

## Author contributions

**Conceptualization:** Yujiao Fang.

**Data curation:** Yuli Qian, Huan Xu, Qiaozhen Li.

**Formal analysis:** Qiaozhen Li.

**Funding acquisition:** Yujiao Fang.

**Investigation:** Qian Zhan.

**Methodology:** Yuli Qian, Jing Diao.

**Project administration:** Yujiao Fang.

**Resources:** Yuli Qian, Qian Zhan.

**Supervision:** Huan Xu, Jing Diao.

**Validation:** Huan Xu, Jing Diao.

**Writing – original draft:** Yujiao Fang, Yuli Qian.

**Writing – review & editing:** Yujiao Fang, Yuli Qian.
